# Wind Turbine Noise and Sleep: Pilot Studies on the Influence of Noise Characteristics

**DOI:** 10.3390/ijerph15112573

**Published:** 2018-11-17

**Authors:** Julia Ageborg Morsing, Michael G. Smith, Mikael Ögren, Pontus Thorsson, Eja Pedersen, Jens Forssén, Kerstin Persson Waye

**Affiliations:** 1 Department of Occupational and Environmental Medicine, Institute of Medicine, University of Gothenburg, 405 30 Gothenburg, Sweden; julia.ageborg.morsing@amm.gu.se (J.A.M.); mikael.ogren@amm.gu.se (M.Ö.); 2 Unit for Experimental Psychiatry, Division of Sleep and Chronobiology, University of Pennsylvania Perelman School of Medicine, Philadelphia, PA 19104, USA; michael.smith@pennmedicine.upenn.edu; 3 Division of Applied Acoustics, Department of Civil and Environmental Engineering, Chalmers University of Technology, 412 96 Gothenburg, Sweden; pontus.thorsson@akustikverkstan.se (P.T.); jens.forssen@chalmers.se (J.F.); 4 Akustikverkstan AB, 531 30 Lidköping, Sweden; 5 Department of Architecture and the Built Environment, Lund University, 221 00 Lund, Sweden; eja.pedersen@arkitektur.lth.se

**Keywords:** wind turbine noise, sleep disturbance, experimental study, amplitude modulation, polysomnography

## Abstract

The number of onshore wind turbines in Europe has greatly increased over recent years, a trend which can be expected to continue. However, the effects of wind turbine noise on long-term health outcomes for residents living near wind farms is largely unknown, although sleep disturbance may be a cause for particular concern. Presented here are two pilot studies with the aim of examining the acoustical properties of wind turbine noise that might be of special relevance regarding effects on sleep. In both pilots, six participants spent five consecutive nights in a sound environment laboratory. During three of the nights, participants were exposed to wind turbine noise with variations in sound pressure level, amplitude modulation strength and frequency, spectral content, turbine rotational frequency and beating behaviour. The impact of noise on sleep was measured using polysomnography and questionnaires. During nights with wind turbine noise there was more frequent awakening, less deep sleep, less continuous N2 sleep and increased subjective disturbance compared to control nights. The findings indicated that amplitude modulation strength, spectral frequency and the presence of strong beats might be of particular importance for adverse sleep effects. The findings will be used in the development of experimental exposures for use in future, larger studies.

## 1. Introduction

Wind is a renewable, sustainable source of power. Gross electricity consumption from wind energy in the European Union (EU) member states increased more than threefold between 2004 and 2014, a trend which can be expected to continue in order to fulfil EU climate goals for 2020 [1]. However, with the increase in wind power, more people will consequently live near wind turbines and are at risk of exposure to wind turbine noise (WTN).

According to the World Health Organization (WHO), an estimated 1.0–1.6 million healthy life years are lost each year due to environmental noise in Western Europe alone [2]. Sleep disturbance is the greatest contributor to this loss, accounting for approximately 900,000 years lost annually. Sleep is a physiological state necessary for maintaining mental and physical well-being [3]. Disturbed sleep can have a negative impact on many aspects of health and wellbeing, including impairment of attention [4], memory consolidation [5,6], neuroendocrine and metabolic functions [7,8], mood [9] and overall quality of life [10]. Night-time noise also affects autonomic functions [11,12], and epidemiological studies have demonstrated that long-term exposure to night-time environmental noise may increase the risk for developing cardiovascular disease [13,14]. 

While sleep disturbance by certain types of environmental noise has been relatively well investigated, particularly transportation noise from rail, air and road traffic [11], there is a relative lack of knowledge regarding the effects of WTN on sleep. Cross-sectional studies in communities with nearby wind farms have demonstrated that WTN causes both annoyance [15,16,17,18,19] and self-reported sleep disturbance [18,19] in a proportion of residents. A recent meta-analysis reported that self-reported high sleep disturbance increased with each A-weighted 10 dB increase in predicted outdoor nocturnal WTN (odds ratio = 1.60, 95% confidence interval: 0.86–2.94) [20]. However, this effect was not statistically significant, and the authors of the meta-analysis concluded that studies with objective measures of sleep and WTN were needed. The results of the meta-analysis were used by the WHO to conclude recently that public health recommendations could not be made for night-time WTN levels, since the quality of evidence was too low [21], assessed via the GRADE approach [22] adopted by the WHO. Low quality evidence in the GRADE approach can be interpreted as “further research being very likely to have an important impact on the certainty of the effect estimate and is likely to change the estimate” [21]. 

At present, effects of WTN have mainly been evaluated using subjective means, and only a few studies have investigated the physiologic response to WTN during sleep. Using wrist actigraphy, Michaud et al. measured sleep of individuals living 0.25–11.22 km from operational wind turbines to examine whether there was an association between objectively measured sleep disturbance and calculated outdoor WTN levels [23]. They found no consistent relationship between sleep disturbance and sound pressure level (SPL) averaged over one year. In another study, Jalali et al. measured sleep using polysomnography (PSG) in participants’ homes, both pre- and post- wind turbine installation and operation [24]. They found no significant differences for any of the measured sleep variables. However, they also did not find any significant differences in SPLs measured in the bedrooms prior to- and after the wind turbines began operating. 

Disturbance from noise depends not only on SPL but also on the characteristics of the noise [25]. The main source of noise from modern wind turbines is aerodynamic noise generated when air passes over the rotor blades [26]. Varying wind speed at different locations in the space swept by the rotor blades can lead to an amplitude modulated sound [27], which may be a possible source of disturbance as it is easily perceived and poorly masked by ambient background noise [15]. WTN is also unpredictable as it varies with wind speed and meteorological conditions [28]. Additionally, WTN is not necessarily attenuated during night-time; in fact, WTN levels may increase during stable atmospheric conditions which occur during the night to a greater extent than during daytime [29,30].

When dose-response curves for WTN levels and annoyance have been compared to previously established dose-response curves for other types of environmental noise (industrial and transportation noise), higher proportions of annoyed residents have been found for WTN at equal SPLs [17,31]. It is likely that several factors other than noise level contribute to response, including respondents’ general attitude towards wind turbines and the experience of procedural fairness or injustice. Furthermore, one possible source of additional annoyance could be that certain characteristics of WTN are more disturbing [31] than those of other types of environmental noise. It is unclear at present whether such acoustical characteristics of WTN are also of relevance for noise-induced effects on sleep.

Because of the need for further research, we implemented a project named Wind Turbine Noise Effects on Sleep (WiTNES), the primary aim of which is a better understanding of causal links between WTN and sleep impairment. Within the project, a method was developed for synthesising WTN, allowing us to generate WTN with no background noise such as traffic, wildlife or meteorological phenomena, and also allowing for manipulation of different acoustical parameters of the noise [32]. Frequency-dependent outdoor to indoor attenuation curves for WTN level were also developed, allowing us to reproduce WTN spectra for indoor locations such as bedrooms, which is relevant for effects on sleep [33]. The present paper presents two pilot studies investigating the effect of wind turbine noise on physiologically measured sleep, conducted with the intention to guide the design and implementation of a larger-scale main study. Of primary interest was aiding the design of sound exposures for the main study. To our knowledge, these are the first studies investigating the effects of wind turbine noise on sleep under controlled laboratory conditions.

## 2. Methods

### 2.1. Experimental Design Overview

Two experimental studies were performed: Study A and Study B. Both studies used a within-subject design, with participants sleeping for five consecutive nights in a sound environment laboratory. Baseline sleep measured during a control night was compared to sleep measured during three nights where participants were exposed to WTN. These exposure nights involved variations of outdoor SPLs and frequency content due to outdoor-indoor filtering, simulating a bedroom with a window being slightly open or closed. Furthermore, within exposure nights there were variations in the acoustic characteristics of WTN.

### 2.2. Experimental Procedure

In order to make the study environment as ecologically valid as possible, the laboratory was outfitted to resemble a typical apartment, with further details and photographs available elsewhere [34]. It contained a combined kitchen and living area, three separate bedrooms and three lavatories. This allowed three individuals to participate concurrently during a given study period, sharing communal areas but sleeping privately. Each of the bedrooms was furnished with a single bed, a desk, a nightstand, chair and lamps. Low frequency noise (≤125 Hz) was introduced through eighty-eight loudspeakers (Sub-Bass modules, Mod. 4 × 10 in, Jbn Development AB, Örnsköldsvik, Sweden) mounted in the ceilings of the bedrooms. Higher frequencies (>125 Hz) were reproduced via two loudspeaker cabinets in the upper corners of the rooms (C115, frequency response 80–20,000 Hz, Martin Audio, High Wycombe, United Kingdom). Lights out was at 23:00 and an automated alarm in the bedrooms woke the participants at 07:00. To ensure there was sufficient time for PSG electrode placement (see below) and relaxation before going to bed, participants were required to arrive at the laboratory by 20:00 each evening. In order to allow participants to adapt to the unfamiliar environment and the PSG equipment used to measure sleep, the first night was a habituation night without exposure to WTN. Data from this night were not used in the analyses. The second night was an exposure-free control night used to measure baseline sleep. During nights 3–5, participants were exposed to WTN. The order of exposure nights was varied between study weeks, however there were only two study weeks in each of the studies and hence the order of nights was not perfectly counterbalanced. A low background noise (18 dB *L*_Aeq_) simulating ventilation noise was played into the bedrooms throughout the study, as otherwise the background level was unnaturally low (≤13 dB *L*_AEq_). Questionnaires were completed by study participants within 15 minutes of waking up. To avoid potential confounders that might affect sleep, participants were prohibited from daytime sleeping, caffeine consumption after 15:00 and alcohol consumption at any time during the studies. 

### 2.3. Polysomnography

Sleep can be broadly classified into two states, rapid eye movement (REM) sleep and non-REM (NREM) sleep. NREM is further divided into three stages which are—in order of increasing depth—N1, N2 and N3 [35]. Different sleep stages have different characteristics in the electroencephalogram (EEG), so we measured physiologic sleep using PSG. We recorded the surface EEG with derivations C3-A2, C4-A1, F3-A2, F4-A1, O1-A2 and O2-A1, electrooculogram and submental electromyogram. Additionally, the electrocardiogram was recorded with two torso electrodes, and pulse, blood oxygen saturation and plethysmogram were recorded using a finger pulse oximeter. Sampling and filter frequencies and placements of electrodes were in line with the American Academy of Sleep Medicine (AASM) guidelines [35]. All data were recorded offline onto an ambulatory PSG device (SOMNOscreen Plus, Somnomedics, Randersacker, Germany). Scoring of the PSG data was performed in line with AASM guidelines [35] by a single experienced sleep technologist who was blind to the study design. EEG arousals, which are abrupt changes in the EEG frequency and sometimes considered indicators of sleep fragmentation [36], were scored as per the American Sleep Disorders Association criteria [37]. Arousals lasting longer than 15 s were classed as awakenings.

Objective sleep variables of interest were sleep onset latency (SOL); total duration and maximum continuous time in stages wake (W), N1, N2, N3 and REM sleep; REM and N3 latency; sleep efficiency (SE); sleep period time (SPT): total sleep time (TST); wakefulness after sleep onset (WASO); timing of first and final awakenings; and the number and frequency of sleep stage changes (SSCs), arousals and awakenings. SOL was the time from lights out until the first non-wake epoch. REM and N3 latencies were the time from sleep onset until the first occurrence of REM or N3 respectively. SPT was the time from sleep onset until the final awakening. WASO was the time spent in W after sleep onset until the final awakening. TST was SPT minus WASO. SE was TST divided by time in bed (TIB, 480 min). SSCs were defined as transitioning from one sleep stage to a lighter stage. Transitions to W were not defined as SSCs but as awakenings. REM sleep was defined as the lightest sleep stage and hence no SSCs could occur from REM. Therefore, SSCs could occur from N3 to N2, N1 or REM, from N2 to N1 or REM and from N1 to REM. 

### 2.4. Questionnaires

In laboratory studies, numerical scales with fixed end points and Likert scales have previously proved capable of detecting the effects of single nights of noise on morning tiredness and perceived sleep quality and depth [38,39], and have been correlated with certain objective sleep measures [40]. Subjective sleep quality was therefore assessed both using an eleven-point numerical scale (anchor points Very poor–Very good) and a five-category Likert scale (Very good; Good; Not particularly good; Poor; Very poor). Nocturnal restoration (anchor points Very tired–Very rested; Very tense–Very relaxed; Very irritated–Very glad) and self-assessed sleep (anchor points Easy to sleep–Difficult to sleep; Better sleep than usual–Worse sleep than usual; Slept deeply–Slept lightly; Never woke–Woke often) were assessed using eleven-point numerical scales. 

Questions pertaining to noise-specific effects on sleep were adapted from recommendations for annoyance questions by the International Commission on the Biological Effects of Noise [41]. An eleven-point numerical scale was used to assess how much participants perceived that WTN disturbed their sleep (anchor points Not at all–Extremely) and four five-category Likert scales were used to investigate whether WTN caused poor sleep, wakeups, difficulties falling back to sleep and tiredness in the morning (Not at all, Slightly, Moderately, Very, Extremely). Also included on the questionnaire were items regarding perceived sleep latency, number of awakenings and whether participants found it difficult or easy to fall asleep following awakenings. The complete questionnaire is presented in the Supplemental Methods. 

### 2.5. Noise Exposure: Study A

Following analysis of field measurements of WTN, three eight-hour night-time exposures of WTN were synthesised (hereafter termed Nights A1, A2 and A3) [32,33]. We varied the noise levels to correspond to different outdoor sound pressure levels in the three nights and used different outdoor-indoor filters to simulate the bedroom window being slightly open (window gap) or closed (Table 1). These resulting indoor noise spectra are given in Supplemental Figure S1. To allow investigation of differential effects of different WTN scenarios, eight periods with different sound character, each 400 s in duration, occurred in each hour of each night. Across the eight hours of the night, the ordering of these sound character periods was balanced in a Latin square so that any period would only follow and precede any other period once. Each hour ended with a 400 s period with no WTN. Based on analysis of existing sound characteristics of WTN [32], the noise scenarios differed in SPL, amplitude modulation (AM) strength (3–4 dB, 7–9 dB, 12–14 dB), rotational frequency of the turbine blades, AM frequency bands (low- or middle-frequency) and the presence or absence of strong beats (Table 2). AM is a rhythmic fluctuation in the noise level, and its calculation is described in detail elsewhere [32]. Beats are in this context defined as strong AM in the frequency range 400–2500 Hz. The spectrum for each sound character period is presented in Supplemental Figure S2.

### 2.6. Noise Exposure: Study B

In Study B the noise level, outdoor-indoor filtering and the frequency band of the amplitude modulation were varied between nights (Table 3). These resulting indoor noise spectra are given in Supplemental Figure S3. Within nights, there were variations in AM strength, rotational frequency and the presence or absence of beats. Unlike Study A, each factor had only two levels, giving a 2 × 2 × 2 factorial design, in order to allow comparison between specific sound characters (see Table 4). Each period was 400 s in duration and each hour ended with a WTN-free 400 s period. The periods were presented in a Latin square as described for Study A. The noise spectrum was kept the same for each sound character period, and is given in Supplemental Figure S4.

### 2.7. Participants

For each of the two studies, six young, healthy participants were recruited via public advertising. Participants in study A (4 women, 2 men) had a mean age of 22.2 years, (standard deviation SD ± 1.3 years) and a mean body mass index (BMI) of 22.6 kgm^−2^ (SD ± 2.4 kgm^−2^). Participants in study B (5 women, 1 man) had a mean age of 24.0 years (SD ± 2.3 years) and a mean BMI of 20.7 kgm^−2^ (SD ± 0.4 kgm^−2^). Participants were screened prior to acceptance with the following exclusion criteria: any self-reported sleep-related disorders; sleeping patterns deviating from the intended sleeping hours in the study; tobacco or nicotine use; dependent on caffeine; regular medication affecting sleep; any self-reported hearing disorders including but not limited to hearing loss, tinnitus and hyperacusis. In order to avoid an increased risk of breathing problems or obstructive sleep apnoea among participants, they were required to have a BMI within the normal range (18.5–24.99 kg/m^−2^). Before acceptance, participants had their hearing tested using pure tone audiometry between 125–8000 Hz to a screening level of 15 dB HL. All participants in both Study A and Study B were classed as being noise sensitive via a single item in the screening questionnaire. All subjects gave their informed consent for inclusion before they participated in the study, and were financially compensated for taking part in the studies. The study was conducted in accordance with the Declaration of Helsinki, and the protocol was approved by the Gothenburg Regional Ethical Review Board (Dnr 974-14).

### 2.8. Statistical Analysis

Statistical analyses were performed in SPSS 22 (IBM Corp., Armonk, NY, USA), employing non-parametric methods. Differences between nights were tested using Friedman tests (within-subject), and if a main effect was found then pairwise comparisons were performed using Wilcoxon signed-rank tests. As a pilot, the primary aim of Study A was not hypothesis testing, but rather to inform on the exposures to be used in future, larger studies [42]. Therefore, analyses were restricted to differences between-nights for PSG variables. In Study B, differences across nights for sound character periods 1–9 across nights were additionally analysed. Time in sleep stages N1, N2, N3 and REM were analysed as fractions of TST. To avoid overlooking any potentially relevant outcomes, a significance level of <0.1 was used, and corrections for multiple comparisons were abdicated. All results should therefore be interpreted with this consideration. Median and interquartile range (IQR) values are reported. 

## 3. Results

### 3.1. Study A: Sleep Micro- and Macro-Structure

**Figure 1:**
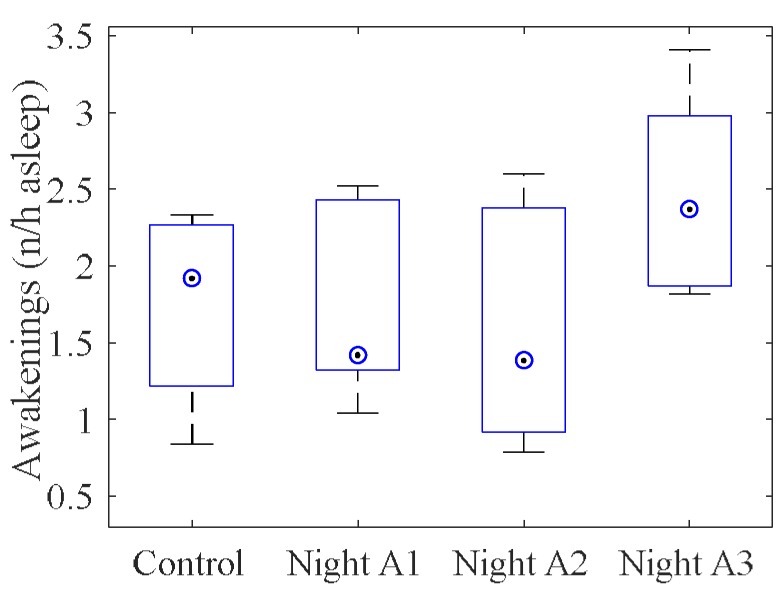
Frequency of awakenings per hour in Study A. Median (◉), interquartile range (boxes) and maximum/minimum values (whiskers).

Mean values of each PSG variable in each study night are given in Supplemental Table S1. One female participant was excluded from analysis of absolute variables as she woke herself up early following two exposure nights. The ratio of events per hour of TST was analysed for cortical reactions: SSCs, arousals, awakenings and combined EEG reactions (both arousals and awakenings together). There was a significant main effect of the frequency of awakenings (χ^2^(df = 3) = 9.0, *p* = 0.029, Figure 1). Awakenings occurred more frequently during nights with indoor noise levels of 34 dB (window closed, Night A3) than in the control night (*p* = 0.046) and nights with 30 or 34 dB with the window slightly open, (Nights A1 and A2, *p* = 0.028 and *p* = 0.028 respectively).

There were no significant main effects between nights for the frequency of arousals, SSCs or combined EEG reactions, or for measures of sleep macrostructure SOL, N3 latency, WASO, time or maximum continuous time in stages W, N1, N2, N3 or REM. 

### 3.2. Study A: Self-Reported Sleep

There was a significant main effect of perceived sleep disturbance by WTN (Table 5) where, relative to the control night, disturbance was greater in Night A2 (*p* = 0.042) and Night A3 (*p* = 0.066). There was also a significant difference in WTN causing tiredness in the morning, with post-hoc tests revealing that Night A3 caused more tiredness in the morning compared to the control night (*p* = 0.059). No significant main effects were found for any of the variables relating to sleep quality, nocturnal restoration, perceived sleep latency or number of recalled awakenings. 

### 3.3. Study B: Sleep Micro- and Macro-Structure

#### 3.3.1. Differences between Nights

**Figure 2:**
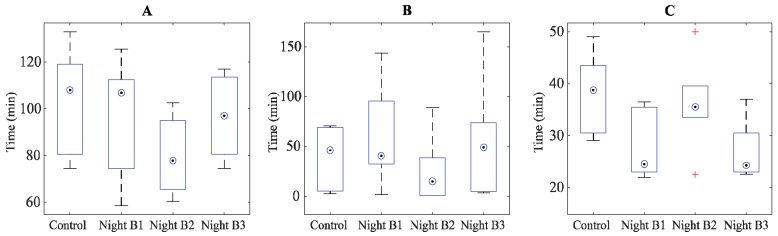
Median (◉), interquartile range (boxes) and maximum/minimum values (whiskers) for objective sleep parameters from Study B. (**A**). Total time in N3. (**B**). Time between sleep onset and first awakening. (**C**). Maximum continuous time in N2 sleep.

Mean values of each PSG variable in each study night are given in Supplemental Table S2. There was a main effect on time spent in N3 (χ^2^(df = 3) = 6.310, *p* = 0.097, Figure 2A), with a significant reduction in N3 sleep in exposure Night B2 compared to the control night (*p* = 0.043) and Night B3 (*p* = 0.046). There was a significant main effect of first awakening (χ^2^(df = 3) = 9.400, *p* = 0.024, Figure 2B), with the first awakening occurring earlier in Night B2 compared to Night B1 (*p* = 0.028) and Night B3 (*p* = 0.028). There was a main effect of maximum continuous time in stage N2 (N2_max_), (χ^2^(df = 3) = 10.200, *p* = 0.017, Figure 2C), where N2_max_ was shorter in Night B1 (*p* = 0.027) and Night B3 (*p* = 0.027) compared to the control night. Furthermore, N2_max_ was shorter in Night B1 (*p* = 0.046) and Night B3 (*p* = 0.028) compared to Night B2. No significant main effects were found for SOL, REM or N3 latencies, total number of SSCs, WASO or SPT. 

#### 3.3.2. Effects of Sound Character Period between Experimental Nights

Main effects were found for percentage of N1 sleep in Period 6, percentage of N3 sleep in Period 4 and for time awake in Period 3 and 7 (Table 6). Participants spent more time awake in Period 7 in Night B1 (*p* = 0.042) and Night B3 (*p* = 0.026) compared to in the control night. However, post-hoc comparisons revealed no significant between-night differences for time awake in Period 3. The percentage of N1 sleep in Period 6 was higher in Night B2 compared to the control night (*p* = 0.028). There was a higher percentage of N1 sleep in Period 6 in Night B2 compared to the control night (*p* = 0.028). The percentage of N3 sleep in Period 4 was significantly lower in Night B2 compared to the control night (*p* = 0.046), Night B1 (*p* = 0.028) and Night B3 (*p* = 0.028). 

Cortical reaction frequencies (arousals, awakenings and SSCs) were calculated for similar sound character periods and analysed to examine whether any specific sound characteristic was of particular importance (Supplemental Figure S5). There were no significant main effects for arousals (*p* = 0.649), awakenings (*p* = 0.197) or SSCs (*p* = 0.191).

#### 3.3.3. Study B: Self-Reported Sleep

Main effects between-nights were found for tiredness in the morning, tension in the morning, difficulties falling asleep, perceived sleep disturbance due to WTN. Furthermore, main effects were found for whether WTN caused poor sleep, awakenings difficulties falling asleep after awakenings or tiredness in the morning (Table 7).

Relative to the control, after Night B1 participants were more tired (*p* = 0.063), had greater difficulty falling asleep (*p* = 0.072) and were more disturbed by WTN (*p* = 0.026). In Night B1, WTN-induced poor sleep (*p* = 0.066), WTN-induced difficulty falling asleep after awakenings (*p* = 0.041) and WTN-induced tiredness (*p* = 0.024) were rated deleteriously compared to the control night. Additionally, perceived disturbance from WTN was greater in Night B1 than Night B2 (*p* = 0.066).

Relative to the control, participants in Night B2 were more disturbed by WTN (*p* = 0.027) and reported more WTN-induced awakenings (*p* = 0.083) and WTN-induced difficulty falling asleep after awakenings (*p* = 0.025).

Relative to the control, participants in Night B3 were more tired (*p* = 0.026), more tense (*p* = 0.041), had more difficulty falling asleep (*p* = 0.027) and were more disturbed by WTN (*p* = 0.027). Furthermore, they indicated more WTN-induced poor sleep (*p* = 0.023), more WTN-induced awakenings (*p* = 0.038), greater WTN-induced difficulty falling asleep after awakenings (*p* = 0.039) and increased WTN-induced tiredness in the morning (*p* = 0.024). Furthermore, tension (*p* = 0.043) and WTN-induced sleep disturbance (*p* = 0.068) were greater following Night B3 than Night B2. WTN-induced tiredness was higher following Night B3 than Night B1 (*p* = 0.083) and Night B2 (*p* = 0.059).

## 4. Discussion

Two studies investigating the effects of nocturnal wind turbine noise on physiologically measured sleep in a laboratory setting have been presented. They were intended to serve as pilot studies prior to a subsequent larger study, and they had the main objective of providing indications of specific sound character of WTN that may be of particular relevance for effects on sleep. Regarding an overall effect of WTN on sleep, there was some evidence that participants had more frequent awakenings, reduced amounts of N3 (“deep”) sleep, reduced continuous N2 sleep, increased self-reported disturbance and WTN-induced morning tiredness in exposure nights with WTN compared to WTN-free nights. 

Furthermore, there was limited evidence from Study B that wakefulness was adversely affected by strong amplitude modulation and lower rotational frequencies, N3 sleep seemed to be adversely affected by higher rotational frequency and strong amplitude modulation and N1 sleep increased with high rotational frequency and beating. However, the current analyses have not accounted for potential interaction effects between sound character periods and exposure night. For instance, it cannot be excluded that an interaction between the exposures used in exposure Night B2 in Study B (50 dB outdoor level with a closed window) and the sound characteristics of Period 4 (high RPM, strong AM, no beats) in the same night is responsible for the observed reduction in N3. 

Awakenings occur spontaneously during sleep, but an increased awakening frequency can disrupt the biorhythm of sleep, causing sleep fragmentation and often resulting in an increase in wakefulness and stage N1 (“light”) sleep with corresponding decreases in deep and REM sleep [38,43]. Deep sleep is believed to be important for nocturnal restoration [44], while N1 may be of little or no recuperative value [45]. Additionally, deep sleep is thought to be important for consolidation of declarative memory, while REM sleep may be important for more implicit memory processes, such as procedural memory [46,47]. While the current studies cannot and do not aim to say anything regarding potential after-effects of the observed changes, the observations of reduced N3, increased N1 and an increased wakefulness under certain sound characteristics of WTN warrants further research.

In Study A, physiologic sleep was generally most impacted during the night with 33.7 dB *L*_AEq,8h,indoor_ closed window and in Study B by nights with low frequency band AM and 32.8 dB *L*_AEq,8h,indoor_ slightly open window. Both cases represent experimental nights with the highest or close to highest SPL in the respective studies, although differences to the lowest WTN levels were at most 4 dB. This provides some small support for the level-dependence for WTN-induced sleep disturbance that has sometimes been seen previously in the field for self-reported measures [19]. In both Studies A and B there were however exposure nights with similarly high noise levels where no effects on sleep were seen, although there were also differences in the AM frequency band or spectral content of the noise due to outdoor-indoor filtering. A possible frequency dependency of WTN-induced effects on sleep should be considered in future work. 

The studies are limited by both the low sample size, and the representativeness of the study population. The low sample size means that only large effect sizes were likely to be detected, even after relaxing the criterion for statistical significance. The participants, being young and healthy individuals with good normal sleep, are not representative of the typical population that may be exposed to WTN at home. However, considering that the aim was to evaluate whether WTN at these levels could have an impact on sleep and whether certain sound characteristics would have a higher impact, the generalisability to a larger population was not the primary concern. Nevertheless, sleep generally deteriorates with increasing age [48], and the prevalence of sleep-related disorders may be around 27% in field settings [49]. It is therefore plausible that the study population represent a particularly robust group, and any WTN-induced effects on sleep may be worse in the field. 

The experimental WTN levels were above the recommended outdoor levels for Sweden [50], although within the recommended outdoor levels for many other countries [51]. The levels were selected to represent worst-case conditions that may occur under unfavourable weather conditions and to increase the likelihood of detecting any effects of WTN despite the low sample size. However, this also means that the findings should not be taken as clear evidence of sleep disturbance due to WTN. The studies were conducted with the aim of providing guidance in the implementation of a larger study, preliminary results of which are available elsewhere [52], and results should be treated accordingly. 

## 5. Conclusions

There were some indications that WTN led to objective sleep disruption, reflected by an increased frequency of awakenings, a reduced proportion of deep sleep and reduced continuous N2 sleep. This corresponded with increased self-reported disturbance. However, there was a high degree of heterogeneity between the two studies presented, precluding firm conclusions regarding effects of WTN on sleep. Furthermore, there was some limited evidence from the second study that wakefulness increase with strong amplitude modulation and lower rotational frequency, the deepest sleep was adversely affected by higher rotational frequency and strong amplitude modulation, and light sleep increased with high rotational frequency and acoustic beating. These findings will be used in the development of noise exposures for a larger-scale sleep study that will implement more naturalistic WTN and use a more representative study population.

## Figures and Tables

**Table 1:** Simulated outdoor and indoor sound pressure levels and frequency filtering used in exposure Nights A1, A2 and A3 in Study A.

Exposure Night	*L*_AEq,8h,outdoor_ (dB)	*L*_AEq,8h,indoor_ (dB)	Filtering
Night A1	40	29.5	Window gap
Night A2	45	34.1	Window gap
Night A3	50	33.7	Window closed

Indoor levels were measured at the pillow position. *L*_AEq,__8h,outdoor_ = Outdoor A-weighted equivalent noise level over the 8 h night-time period. *L*_AEq,8h,indoor_ = Indoor A-weighted equivalent noise level over the 8 h night-time period.

**Table 2:** Overview of the 400 s sound character periods within each hour in Study A.

Period	*L*_AEq_ Relative to 8-h Level (dB)	Rotational Frequency (rpm)	AM Strength	AM Frequency Band (Hz)	Beats
1	−2.5	15	7–9 dB	500–2000	No
2	-	15	7–9 dB	500–2000	No
3	+2.5	15	7–9 dB	500–2000	No
4	-	13	7–9 dB	80–315	No
5	-	17	12–14 dB	500–2000	Yes
6	-	14	3–4 dB	500–2000	No
7	-	15	12–14 dB	500–2000	No
8	-	18	12–14 dB	500–2000	Yes
9	No WTN				

Sound character was varied in level, turbine rotational frequency, amplitude modulation (AM) strength, AM frequency band and presence or absence of strong beats. Periods 1–8 were counterbalanced across the 8 night-time hours. Period 9 was always the final 400 s of each hour. L_AEq_ = A-weighted equivalent noise level.

**Table 3:** Outdoor and indoor sound pressure levels, frequency filtering and AM frequency bands used in exposure Nights B1, B2 and B3 in Study B.

Exposure Night	*L*_AEq,8h,outdoor_ (dB)	*L*_AEq,8h,indoor_ (dB)	Filtering	AM Frequency Band (Hz)
Night B1	45	32.8	Window gap	160–500
Night B2	45	32.8	Window gap	80–315
Night B3	50	30.4	Window closed	80–315

Indoor levels were measured at the pillow position. *L*_AEq,8h,outdoor_ = Outdoor A-weighted equivalent noise level over the 8 hour night-time period.

**Table 4:** Overview of the 400 s sound character periods within each hour in Study B.

Period	Rotational Frequency (rpm)	AM Strength	Beats
1	13	3–4 dB	No
2	17	3–4 dB	No
3	13	12–14 dB	No
4	17	12–14 dB	No
5	13	3–4 dB	Yes
6	17	3–4 dB	Yes
7	13	12–14 dB	Yes
8	17	12–14 dB	Yes
9	No WTN		

Sound character was varied in turbine rotational frequency, amplitude modulation (AM) strength, and presence or absence of strong beats. Periods 1–8 were counterbalanced across the 8 night-time hours. Period 9 was always the final 400 s of each hour.

**Table 5:** Self-reported sleep variables where a main effect of night was found in Study A.

Sleep Measure	Median (IQR)				χ^2^	*p*-Value
	Control	Night A1	Night A2	Night A3		
Sleep disturbance by WTN (0 = Not at all, 10 = Extremely)	0 (0–0.75)	0 (0–2.5)	1.5 (0.75–4)	2.5 (0–4.75)	7.227	0.065
WTN cause tiredness in the morning (Not at all = 1; Extremely = 5)	1 (1–1)	1 (1–2.25)	1 (1–2.25)	2 (1–2.25)	6.400	0.094

IQR = Interquartile range.

**Table 6:** Objective sleep variables where a main effect of WTN sound character period was found in Study B.

Sleep Measure	Period	Median (IQR)				χ^2^	*p*-Value
		Control	Night B1	Night B2	Night B3		
Time awake (min)	3 ^a^	1 (0.50–1.63)	0.75 (0.50–1.25)	1.75 (0.75–5.13)	2 (0.88–2.88)	7.000	0.072
	7 ^b^	0.75 (0.38–1.13)	1.75 * (1.50–2.0)	1.25 (0.50–2.75)	6.63 * (5.74–7.52)	8.509	0.037
N1 (%)	6 ^c^	6.63 (5.74–7.52)	6.37 (0.71–13.84)	11.32 * (8.47–15.64)	4.69 (1.81–5.27)	11.400	0.010
N3 (%)	4 ^d^	26.77 (21.24–29.41)	29.12 (13.60–33.02)	4.60 *^,^† (0–13.58)	27.22 (18.72–32.89)	10.900	0.014

^a^ 13 rpm, strong AM, no beats; ^b^ 13 rpm, strong AM, beats; ^c^ 17 rpm, weak AM, beats; ^d^ 17 rpm, strong AM, no beats. Significant (*p* < 0.05) post-hoc differences to the control night are denoted *. Significant (*p* < 0.05) post-hoc differences to both Night B1 and Night B3 are denoted †. IQR = Interquartile range.

**Table 7:** Self-reported sleep variables in Study B.

Sleep Measure	Median (IQR)				χ^2^	*p*-Value
	Control	Night B1	Night B2	Night B3		
Sleep quality (Very good = 0, Very poor = 10)	3 (2.75–6.50)	4.5 (2–5.5)	4.5 (1–7.5)	6 (4.25–6.25)	0.911	ns
Verbal sleep quality (Very good = 1, Very poor = 5)	2 (2–2.25)	2 (1.75–4)	2 (1–2.75)	3 (2–3.25)	3.692	ns
Very rested (0)–Very tired (10)	2.5 (1.75–3.25)	5.5 * (1.75–6.25)	2.5 (1.5–6.75)	5.5 * (4–7)	9.367	0.025
Very relaxed (0)–Very tense (10)	3 (2.5–3.5)	4.5 (1–6)	3 (1–4.25)	5.5 *† (4.5–7)	8.625	0.035
Very glad (0)–Very irritated (10)	2 (0.75–4.75)	3.5 (1.75–7)	4 (1–4.5)	5.5 (3.75–6.25)	5.308	ns
Time to fall asleep (min)	15 (8.75–22.5)	27.5 (15.5–38.75)	15 (8.75–46.25)	25 (16.25–42.50)	3.808	ns
Estimated number of wakeups (n)	2 (2–3)	2 (2–4.25)	2.5 (1.75–4)	3 (1.75–3)	0.796	ns
Easy to sleep (0)–Difficult to sleep (10)	3 (0.75–4)	6 * (2.75–8)	2.5 (1–7.25)	6.5 * (4.25–8)	8.793	0.032
Slept better than usual (0)–Worse than usual (10)	5 (4.25–7.25)	6 (4.75–8.25)	5 (2.75–7.5)	7 (6–8.25)	3.982	ns
Deep sleep (0)–Light sleep (10)	3 (2.5–4.25)	6 (2–7.5)	3.5 (1.75–6.75)	6 (3–7.25)	3.911	ns
Never woke (0)–Woke often (10)	6.5 (5–7.25)	4 (2.75–9)	4 (3.25–5)	6 (2.75–7)	0.661	ns
Sleep disturbance by WTN (0 = Not at all, 10 = Extremely)	0 (0–0.25)	2.5 *† (2–7.25)	2.5* (1–4.5)	6 *‡† (3.5–6.25)	14.722	0.002
WTN cause poor sleep (Not at all = 1, Extremely = 5)	1 (1–1)	2 * (1–3.25)	2 (1–3)	3 * (2–3)	10.432	0.015
WTN cause awakenings (Not at all = 1, Extremely = 5)	1 (1–1.25)	1.5 (1–3.25)	1.5 * (1–2.25)	2.5 * (1.75–3.25)	9.250	0.026
WTN cause difficulties falling back to sleep (Not at all = 1, Extremely = 5)	1 (1–1)	2.5 * (1.75–4)	2 * (1.75–2)	3 * (1.75–3.25)	9.889	0.020
WTN cause tiredness in the morning (Not at all = 1, Extremely = 5)	1 (1–1.25)	2 * (2–4)	2 (1.75–3.25)	3 *† (2.75–4)	15.125	0.002

Sleep quality was coded such that the scales are in the same direction as for other items, i.e., a higher value indicates worse sleep. *p*-values relate to tests of main effects. ns = not significant (α = 0.1). Significant (*p* < 0.1) post-hoc tests are denoted * (compared to control night); ‡ (compared to Night B1); † (compared to Night B2). IQR = Interquartile range.

## Supplementary Materials

The following are available online at http://www.mdpi.com/1660-4601/15/11/2573/s1. Morning questionnaire. Figure S1: Indoor average spectra across the full 8-hour exposure period for each WTN night in Study A. Figure S2: Outdoor spectrum (40 dB *L*_AEq,8h_) for each sound character period in Study A. Figure S3: Indoor average spectra across the full 8-hour exposure period for each WTN night in Study B. Figure S4: Outdoor spectrum (45 dB *L*_AEq,8h_) for each sound character period in Study B. Table S1: Mean and standard deviation (SD) of sleep macro- and micro-structure data for each night in Study A. Table S2: Mean and standard deviation (SD) of sleep macro- and micro-structure data for each night in Study B. Figure S5: Median, interquartile range, maximum/minimum values and outliers for cortical reaction frequency across periods of different character WTN. A) Arousals. B) Awakenings. C) Sleep stage changes.

## Glossary

AM	Amplitude modulation. A time-varying increase and decrease in sound pressure level, which can vary for different frequencies of the same sound signal
A-Weighting	Frequency weighting filter applied to a sound measurement to mimic the frequency-dependence of human hearing
dB	Decibel, relative to the threshold of human hearing (2 × 10^−5^ Pa)
EEG	Electroencephalogram
*L*_AEq_	A-weighted equivalent continuous sound pressure level, expressed in decibels. Can be considered the “average” of a time-varying sound pressure level over a specified period
*L*_AEq,8h,indoor_	A-weighted equivalent continuous indoor sound pressure level over 8 h
*L*_AEq,8h,outdoor_	A-weighted equivalent continuous outdoor sound pressure level over 8 h
NREM	Non-rapid eye movement
PSG	Polysomnography
SSC	Sleep stage change
REM	Rapid eye movement
WHO	World Health Organization
WTN	Wind turbine noise
