# Supplementary material for: Wind Turbine Noise and Sleep: Pilot Studies on the Influence of Noise Characteristics
Source: Int J Environ Res Public Health. 2018 Nov 17;15(11):2573. doi: 10.3390/ijerph15112573 (PMC6266820; doi:10.3390/ijerph15112573)
Supplement: Supplementary file 1 [file ijerph-15-02573-s001.pdf]

Wind turbine noise and sleep: Pilot studies on the influence of noise characteristics

Supplemental Material

Julia Ageborg Morsing , Michael G. Smith, Mikael Ögren, Pontus Thorsson , Eja Pedersen, Jens Forssén and Kerstin Persson Waye*

*Corresponding author: Kerstin Persson Waye, kerstin.persson.waye@amm.gu.se

Supplemental Methods

The following section includes the complete morning questionnaire and noise spectra for the experimental wind turbine noise.

Morning questionnaire

The original questionnaires were administered in Swedish. Below is the English translation of the questionnaire, with identical layout and formatting as used in the paper questionnaires in the study.

Questions to answer in the morning

Answer the questions within 15 minutes of awakening at 7:00 in the morning

Note that this form is two-sided

1. **How would you rate your sleep quality during the night?** Circle the appropriate number.

Very good 0 1 2 3 4 5 6 7 8 9 10 Very bad

Also give your answer on the following verbal scale

2. **How would you rate your sleep quality during the night?**

[ ] Very good
[ ] Rather good
[ ] Not particularly good
[ ] Bad
[ ] Very bad

**How are you feeling right now?**	*Circle the appropriate number*												
3. Very rested	0	1	2	3	4	5	6	7	8	9	10	Very tired
4. Very relaxed	0	1	2	3	4	5	6	7	8	9	10	Very tense
5. Very irritated	0	1	2	3	4	5	6	7	8	9	10	Very glad

6. **How long did it take you to fall asleep last night?** Minutes:__________

7. **How many times do you estimate that you woke up during the night before the morning alarm?** [ ] Did not wake Woke ____ times

8. **Did you have difficulty falling back to sleep after an awakening?** [ ] Did not wake [ ] No [ ] Yes

How was your experience of the night and your sleep?													Circle the appropriate number
9.	Easy to fall asleep	0	1	2	3	4	5	6	7	8	9	10	Difficult to fall asleep
10.	Better sleep than usual	0	1	2	3	4	5	6	7	8	9	10	Worse sleep than usual
11.	Deep sleep	0	1	2	3	4	5	6	7	8	9	10	Shallow sleep
12.	Never woke up	0	1	2	3	4	5	6	7	8	9	10	Woke up often

13. **How disturbed was your sleep by noise from wind turbines during the night?**
Circle the appropriate number

Not at all 0 1 2 3 4 5 6 7 8 9 10 Extremely

Do you think that noise during the night disturbed your sleep so that you:

		Not at all	Slightly	Moderately	Very	Extremely
14.	slept badly?	[ ]	[ ]	[ ]	[ ]	[ ]
15.	were awoken?	[ ]	[ ]	[ ]	[ ]	[ ]
16.	had difficulty falling asleep?	[ ]	[ ]	[ ]	[ ]	[ ]
17.	felt tired in the morning?	[ ]	[ ]	[ ]	[ ]	[ ]

Noise spectra

The following figures show the outdoor WTN spectra for each sound character period in each study (Figure S1 and S3), and the average whole night indoor spectrum for each exposure night in each study (Figure S2 and S4). Although there were differences in turbine rotational frequency, amplitude modulation (AM) strength and the presence of acoustic beating in Study B, the spectrum was identical across the different sound character periods, and therefore only a single outdoor spectrum is presented. Similarly, although there were differences in the AM frequency range between Night B1 and Night B2 in study B, the overall spectra were identical.

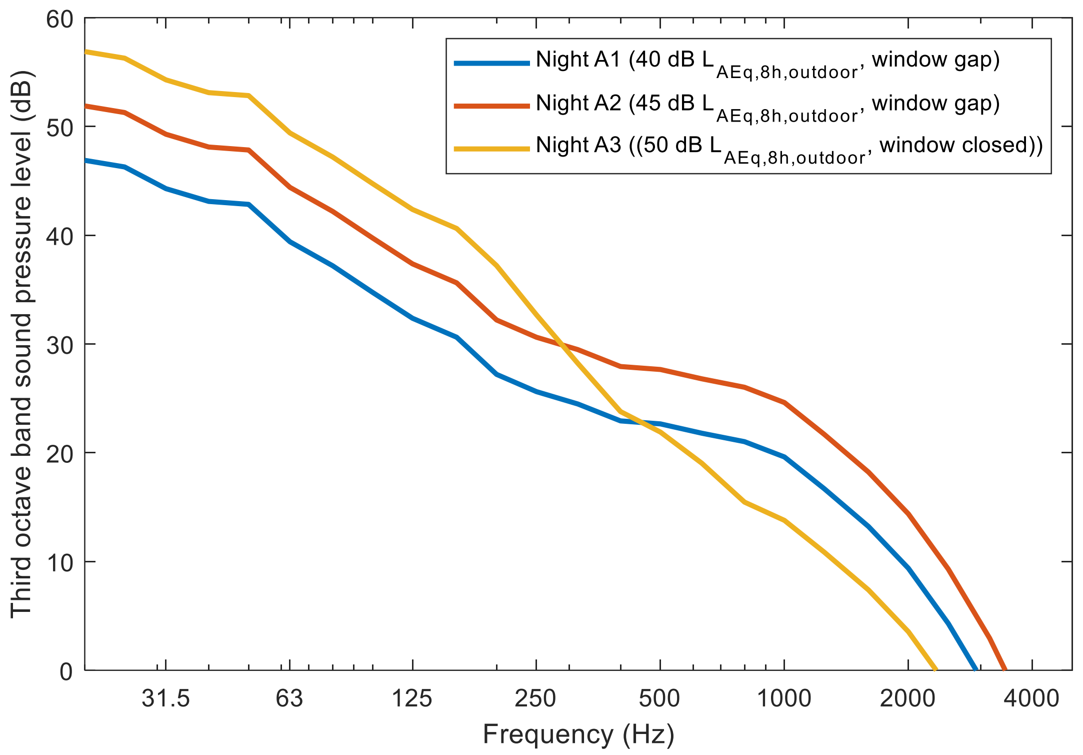

Figure S1 Indoor average spectra across the full 8-hour exposure period for each WTN night in Study A.

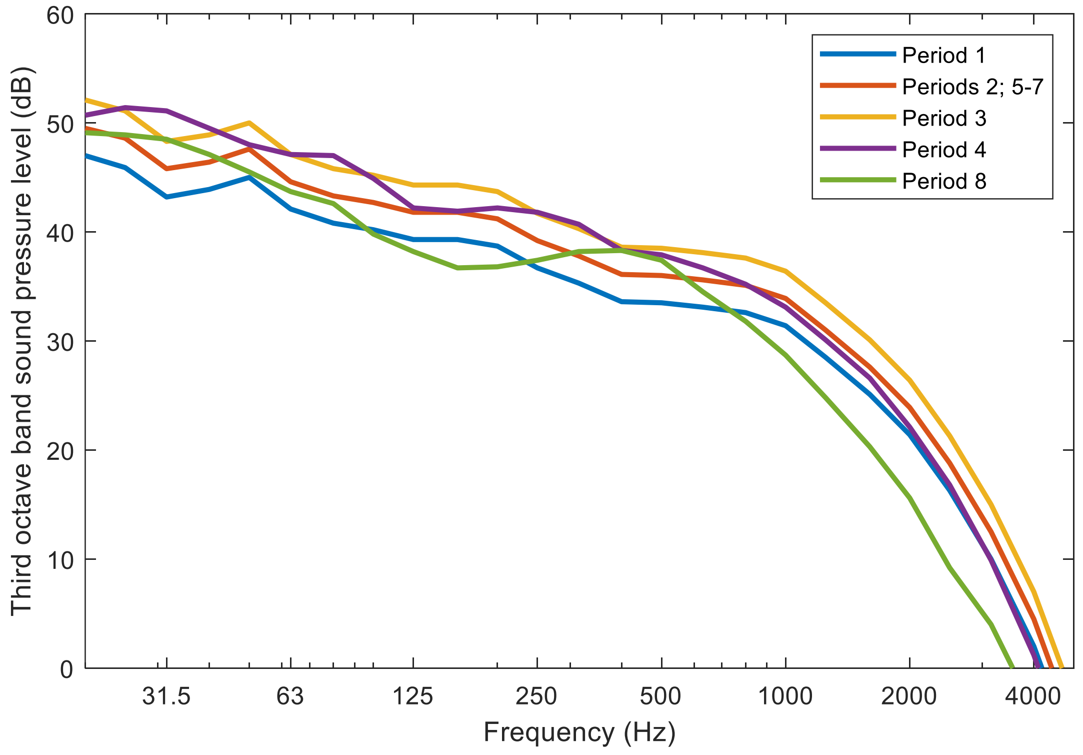

Figure S2 Outdoor spectrum (40 dB $L_{AEq,8h}$) for each sound character period in Study A. Spectra were identical for Periods 2, 5, 6 and 7.

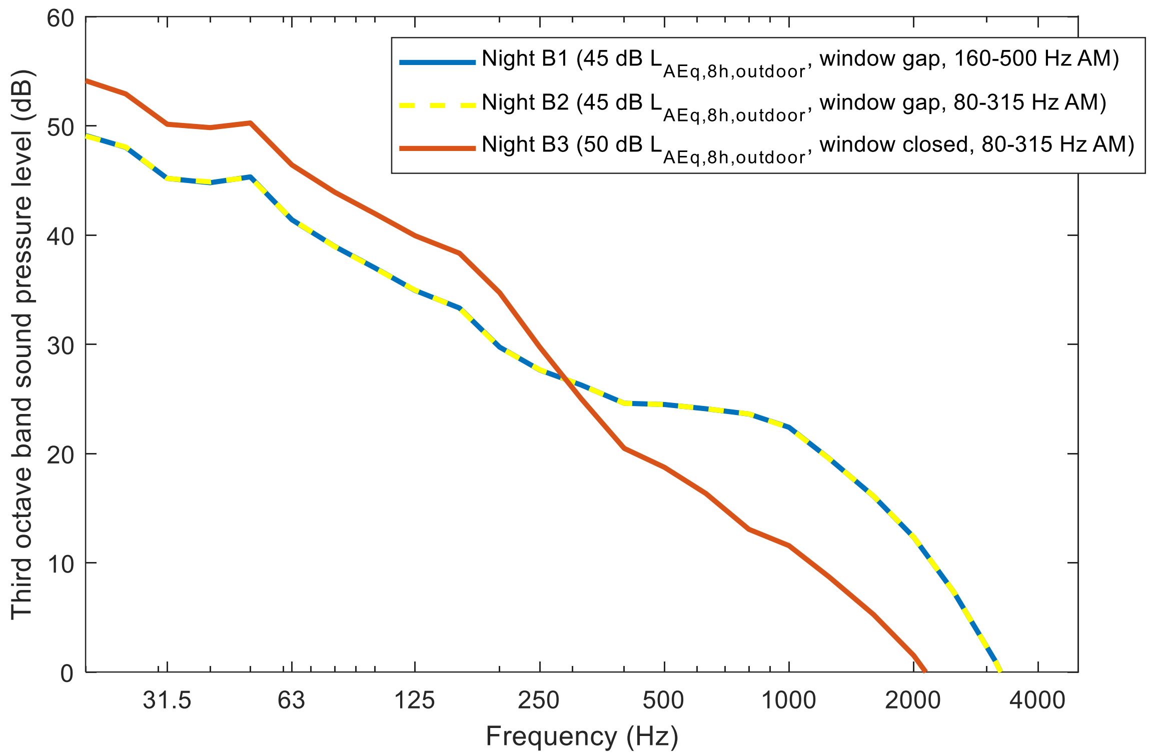

Figure S3 Indoor average spectra across the full 8-hour exposure period for each WTN night in Study B. Spectra were identical for Nights B1 and B2.

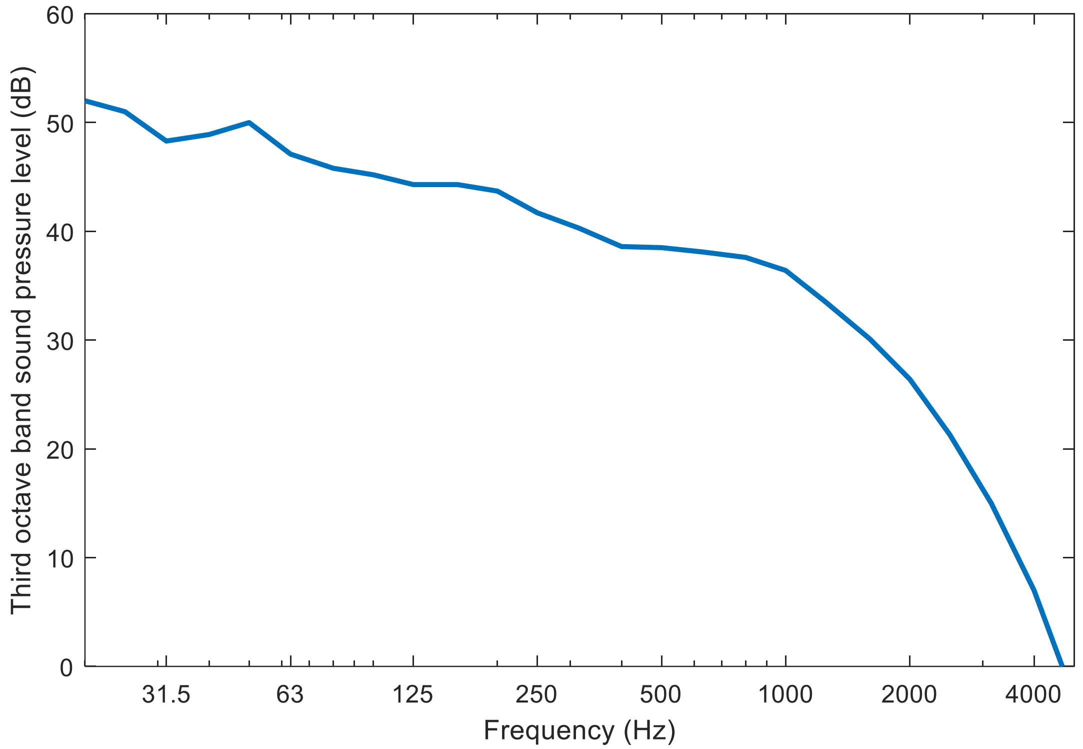

Figure S4 Outdoor spectrum (45 dB $L_{AEq,8h}$) for each sound character period in Study B.

Supplemental Results

Means of objectively measured sleep macro- and microstructure data are given for Study A and Study B in Table S1 and Table S2 respectively.

Data from Study B for arousal, awakening and sleep stage change reaction frequency across periods of different character wind turbine noise are given in Figure S5.

Table S1 Mean and standard deviation (SD) of sleep macro- and micro-structure data for each night in Study A. Noise levels indicate outdoor noise levels, 'gap' or 'closed' indicates the outdoor-indoor window filter used. SOL: Sleep onset latency; TST: Total sleep time; SPT: Sleep period time; SSC: Sleep stage changes.

Variable	Control		A1 (40 dB, gap)		A2 (45 dB, gap)		A3 (50 dB, closed)	
	Mean	SD	Mean	SD	Mean	SD	Mean	SD
SOL (min)	23.3	20.6	20.4	13.2	16.0	7.2	13.7	8.0
TST (min)*	425.9	32.5	444.9	13.8	429.2	32.4	448.6	8.4
SPT (min)*	453.3	21.1	455.4	13.6	460.8	6.6	466.9	8.8
Sleep efficiency (%)	90.0	6.8	93.2	2.5	90.3	6.4	93.6	1.6
REM (% of TST)	22.6	4.5	22.9	5.3	21.7	4.9	23.3	1.5
N1 (% of TST)	8.8	2.5	7.7	1.8	9.0	2.5	7.3	1.2
N2 (% of TST)	47.7	5.2	48.9	7.5	46.8	7.0	47.7	4.9
N3 (% of TST)	20.9	3.4	20.6	6.7	22.5	4.6	21.7	3.7
Maximum continuous wake (min)	24.4	21.1	19.3	12.0	25.9	23.1	14.1	7.5
Maximum continuous REM (min)	20.7	8.1	17.9	3.7	17.0	6.7	20.0	8.5
Maximum continuous N1 (min)	4.5	1.3	4.2	1.2	3.7	1.4	3.0	0.8
Maximum continuous N2 (min)	30.7	10.6	34.3	9.0	28.5	4.3	31.0	6.8
Maximum continuous N3 (min)	37.9	10.4	32.6	10.6	37.5	12.1	37.8	7.8
REM latency (min)	82.9	15.7	69.1	13.3	111.8	30.9	77.2	12.1
N3 latency (min)	18.5	13.7	14.3	3.1	28.5	36.1	13.7	5.3
First awakening (min)	33.1	30.3	54.7	59.9	30.4	27.2	47.0	28.1
Arousals (n/h asleep)	6.23	1.39	5.02	0.64	5.62	1.92	6.30	2.39
Awakenings (n/h asleep)	1.75	0.63	1.69	0.63	1.58	0.75	2.47	0.66
Combined EEG reactions (n/h asleep)	7.98	1.50	7.31	1.90	7.87	2.94	7.49	1.10
SSC (n/h asleep)	3.01	0.52	3.22	0.99	3.19	1.06	3.01	0.67

* One female participant was excluded as she woke herself up early following two exposure nights.

Table S2 Mean and standard deviation (SD) of sleep macro- and micro-structure data for each night in Study B. Noise levels indicate outdoor noise levels, 'gap' or 'closed' indicates the outdoor-indoor window filter used. AM: amplitude modulation; SOL: Sleep onset latency; TST: Total sleep time; SPT: Sleep period time; SSC: Sleep stage changes.

Variable	Control		B1 (40 dB, gap, 160-500 Hz AM)		B2 (45 dB, gap, 80-315 Hz AM)		B3 (50 dB, closed, 80-315 Hz AM)	
	Mean	SD	Mean	SD	Mean	SD	Mean	SD
SOL (min)	10.3	8.4	17.5	10.6	17.0	11.4	21.3	25.5
TST (min)	455.2	9.2	447.5	14.7	442.7	9.9	440.8	34.4
SPT (min)	466.3	9.6	461.7	12.2	459.3	6.4	456.5	24.3
Sleep efficiency (%)	94.8	1.9	93.2	3.1	92.2	2.1	91.8	7.2
REM (% of TST)	21.3	2.6	20.7	2.8	21.3	3.3	20.0	1.0
N1 (% of TST)	7.2	2.8	7.9	3.2	8.5	2.7	7.6	3.1
N2 (% of TST)	48.6	3.7	49.8	4.8	52.1	5.4	50.5	4.0
N3 (% of TST)	22.8	4.9	21.7	5.3	18.0	3.7	22.0	4.0
Maximum continuous wake (min)	13.3	8.1	18.3	9.2	19.5	8.1	22.0	24.9
Maximum continuous REM (min)	15.8	4.0	16.8	4.7	14.7	4.8	16.3	7.1
Maximum continuous N1 (min)	3.3	1.3	3.5	1.2	5.1	2.5	3.4	1.0
Maximum continuous N2 (min)	38.3	8.0	27.7	6.6	36.1	9.0	26.9	5.7
Maximum continuous N3 (min)	40.2	10.3	34.8	10.2	32.9	16.9	31.0	8.9
REM latency (min)	79.2	21.4	66.3	8.2	71.0	14.5	65.7	11.7
N3 latency (min)	17.3	5.0	18.3	8.2	16.9	7.6	16.8	5.0
First awakening (min)	39.8	30.0	58.8	51.4	26.3	34.7	57.3	59.6
Arousals (n/h asleep)	7.53	3.12	6.82	2.14	7.54	2.18	7.37	2.04
Awakenings (n/h asleep)	1.36	0.55	1.50	0.78	1.70	0.56	1.54	0.92
Combined EEG reactions (n/h asleep)	8.89	3.31	9.04	2.47	9.07	2.35	8.36	2.87
SSCs (n/h asleep)	3.28	0.74	3.40	0.88	3.27	0.86	3.24	1.11

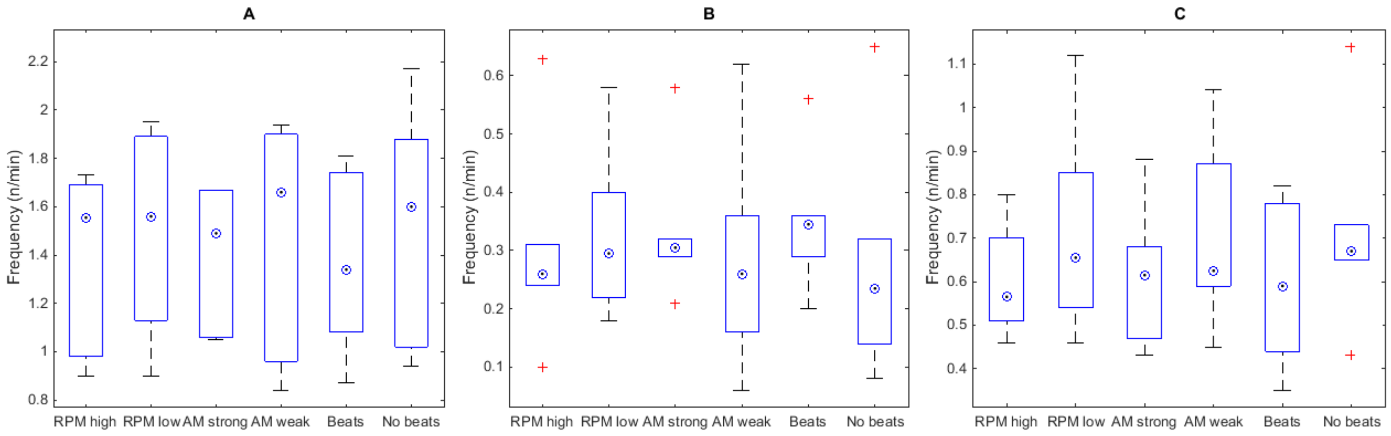

Figure S5 Median (⦿), interquartile range (boxes), maximum/minimum values (whiskers) and outliers (+) for cortical reaction frequency across periods of different character WTN. **A.** Arousals. **B.** Awakenings. **C.** Sleep stage changes.
